# Urothelial Carcinoma of the Bladder With Primary Metastasis to the Brain: A Case Report and Literature Review

**DOI:** 10.7759/cureus.27587

**Published:** 2022-08-01

**Authors:** Madhav Sankhyan, Evan M Anderson, Jorge F Urquiaga, Jakob T Hockman, Ruchy Aggarwal, Najib E El Tecle, Philippe J Mercier

**Affiliations:** 1 Neurological Surgery, Saint Louis University School of Medicine, St. Louis, USA; 2 Neurological Surgery, Stony Brook University, Stony Brook, USA

**Keywords:** transitional cell carcinoma, isolated brain metastasis, metastatic brain tumors, neurosurgery oncology, urothelial malignancy, cerebral metastasis

## Abstract

Brain metastases are the most common type of brain tumor in adults, commonly arising from primary tumor sites of the lung, breast, skin (melanoma), colon, and kidney. Isolated central nervous system (CNS) metastasis arising from urothelial carcinoma (UC) is a rare presentation yielding a poor prognosis.

A 71-year-old male patient with a history of urothelial carcinoma, treated one year prior with partial cystectomy and adjuvant gemcitabine and cisplatin (GC) therapy, presented with worsening neurological symptoms, including progressively worsening dizziness, shuffling gait, drifting, expressive aphasia, and confusion. MRI revealed a left frontal 4.0 x 3.6 cm brightly contrast-enhancing tumor with possible hemorrhage, extensive vasogenic edema, and moderate mass effect. An additional smaller right cerebellar lesion was also noted. Outpatient CT of his chest, abdomen, and pelvis revealed no evidence of other malignant sites. He ultimately underwent a left craniotomy with a total resection of his left frontal mass. Pathological examination revealed a urothelial primary. Post-operative MRI revealed complete resection of the left frontal mass and the patient was discharged with no neurologic deficits on exam.

In many cases, brain metastases may present years later following initial therapy of UC as the CNS may act as a sanctuary site during systemic chemotherapy. Chemotherapeutics such as gemcitabine with better penetration of the blood-brain barrier may be beneficial in delaying the onset of these metastases.

## Introduction

Urothelial carcinoma (UC), also known as transitional cell carcinoma (TCC), accounts for more than 90% of all bladder cancers in the western world and is the fifth most common malignancy worldwide, comprising 2% of all reported malignancies [1-5]. Ninety percent of urothelial carcinomas present as bladder tumors, with approximately 5-10% presenting as upper tract urothelial carcinomas (UTUC) that involve the renal pelvis, ureters, or calyces, although UC can arise anywhere along the urinary tract [2,6,7].

UC can theoretically metastasize to any organ, although the most common sites of metastasis include lymph nodes, liver, lung, peritoneum, and bone, while a UC metastasis to the brain is a rare occurrence [8]. Distant metastases are estimated to occur in 10-29% of patients with bladder cancer, and 67% of patients have evidence of metastases on autopsy [1,4,5]. In contrast, 1-7% of patients are reported to have brain metastases from primary UC, although reliable data on this prevalence is scarce and estimates vary in part due to the rarity of reported cases [1,4,5]. Thus, routine neurological imaging for intracranial metastases is often not recommended [6].

The occurrence of brain metastases confers a poor prognosis, with some authors estimating the median survival of treated patients to be 2-4 months while the median survival of untreated patients ranges from a couple days to 1.75 months [5,6]. Additionally, the estimated median time to brain metastasis in patients with UC was 10 months [6]. The rare occurrence and poor prognosis of brain metastases in UC contribute to the uncertain management and treatment protocol for these patients, which we hope to help elucidate through the contribution of this report. Here we present a rare case of brain metastasis from a primary urothelial carcinoma and discuss treatment options based on the current literature.

## Case presentation

Our patient is a 71-year-old male with a medical history of bladder cancer who presented to our institution as a transfer for evaluation of a newly discovered brain lesion. The patient had been having progressively worsening dizziness, shuffling gait, drifting, expressive aphasia, and confusion for the past few weeks. The outside hospital reported MRI findings of a large, left-frontal, contrast-enhancing lesion, as well as a smaller, right-cerebellar lesion surrounded by vasogenic edema. They began levetiracetam and dexamethasone and then transferred the patient to our institution for further evaluation. On arrival, the patient stated his symptoms had improved since the administration of steroids. He denied current headaches, confusion, nausea and vomiting, weakness, numbness, tingling, or seizures.

The patient had a known history of urothelial carcinoma. One year prior to admission, he underwent four cycles of neoadjuvant chemotherapy with cisplatin and gemcitabine followed by partial cystectomy and pelvic node dissection for a high-grade, T2N3, papillary urothelial carcinoma of the bladder.

The patient was alert and oriented to person, place, time, and situation. His neurological exam was unremarkable except for the presence of right-sided dysmetria.

**Figure 1:**
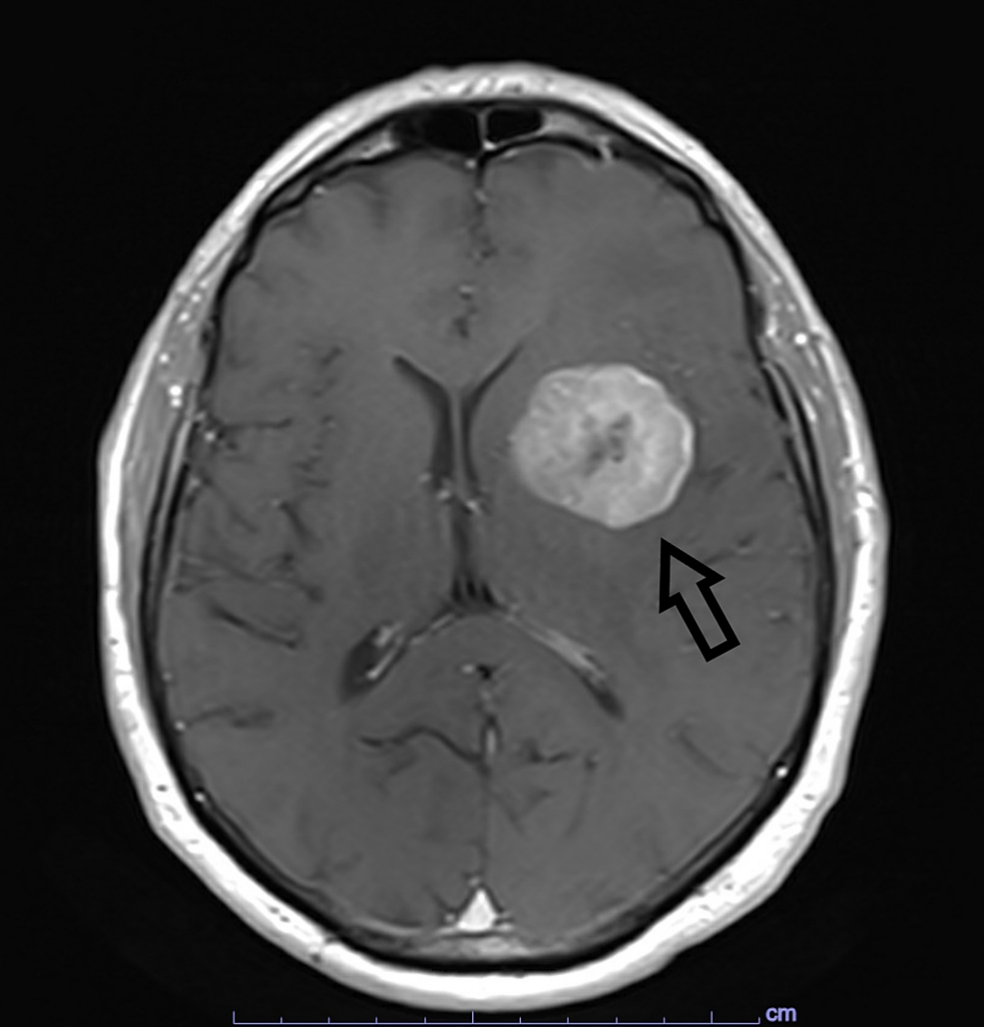
Pre-operative, T1-weighted, axial MRI image with contrast demonstrating left frontal lesion. Arrow: left frontal lesion

**Figure 2:**
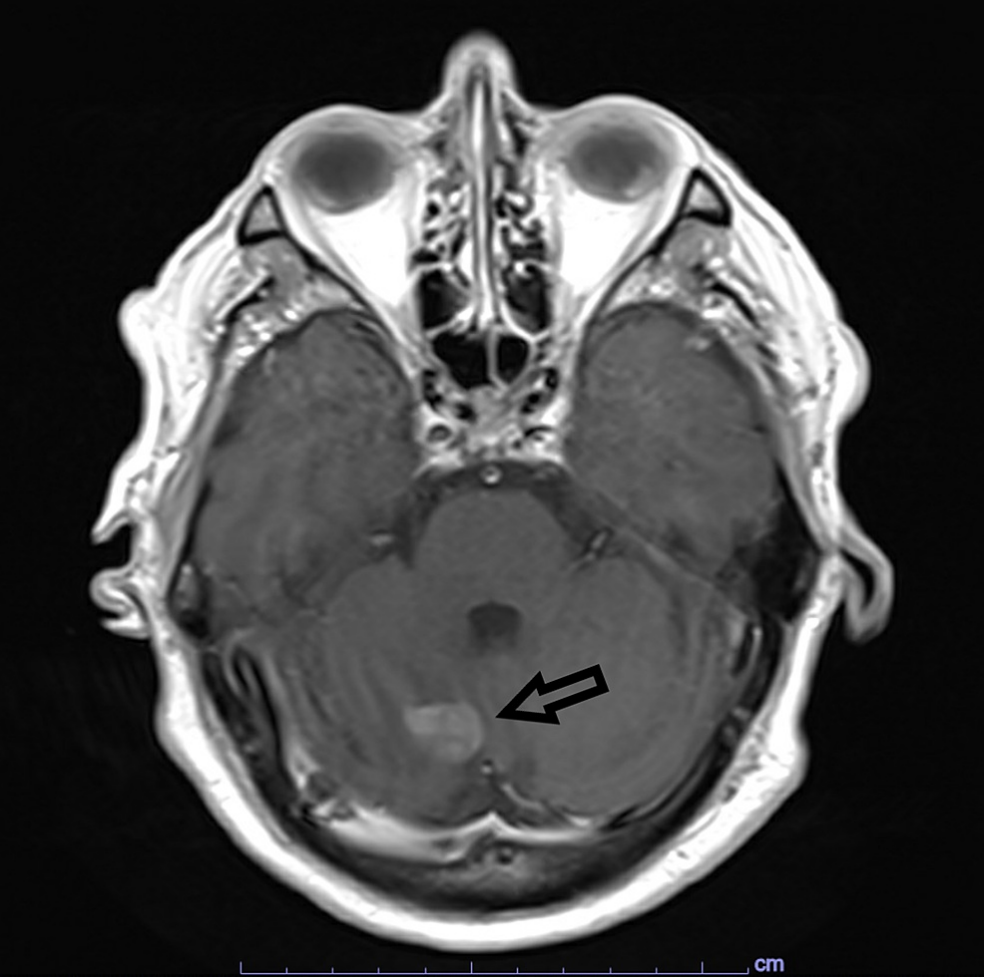
Pre-operative, T1-weighted, axial MRI image with contrast demonstrating right cerebellar lesion. Arrow: right cerebellar lesion

His labs on presentation were only remarkable for a glucose of 373 mg/dl. An outpatient CT of his chest, abdomen, and pelvis with contrast revealed no evidence of malignancy. The MRI from the outside hospital documented a left frontal 4.0 x 3.6 cm brightly contrast-enhancing tumor with possible hemorrhage, extensive vasogenic edema, and moderate mass effect. An additional smaller right cerebellar lesion was also noted. There was a 6-mm left to right midline shift on the coronal image. An in-house MRI was performed that demonstrated similar findings (Figures 1, 2). A subsequent functional MRI revealed none of the visual, language, or motor centers involved in the left frontal or right cerebellar masses. During the exam, the patient was unable to follow the instructions of a picture naming task.

**Figure 3:**
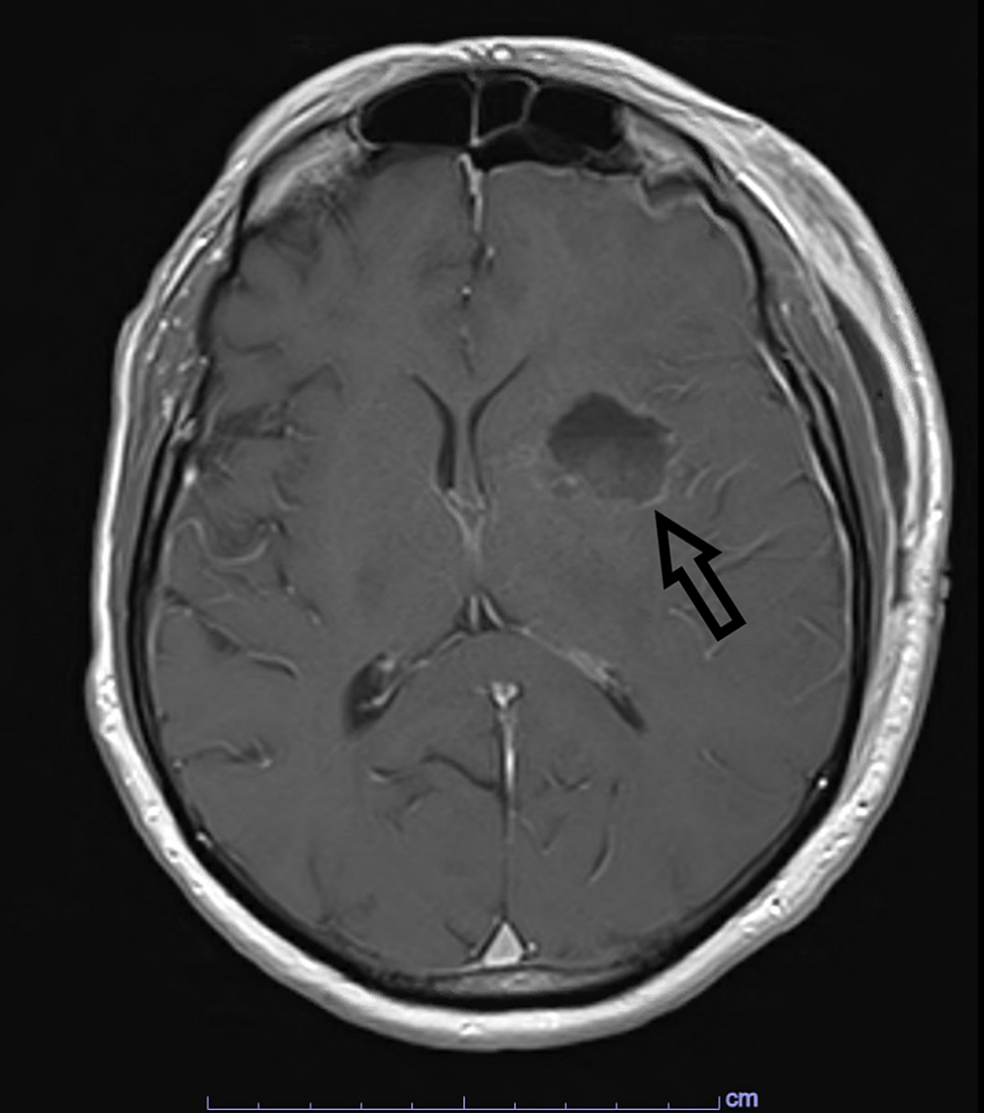
Post-operative, T1-weighted, axial MRI image with contrast demonstrating resected left frontal lesion. Arrow: resection cavity of the left frontal lesion

The patient ultimately underwent a left craniotomy with total resection of the left-frontal mass. A postoperative MRI demonstrated complete resection of the left frontal mass with no interval change in the right cerebellar mass (Figure 3). There were no intraoperative or postoperative complications. Pathology confirmed a metastatic carcinoma consistent with a urothelial primary. The patient was discharged home with no neurologic deficits on exam. Further management was deferred to his medical oncologist, who recommended stereotactic radiosurgery but no systemic therapy. Unfortunately, the patient was lost to follow-up and further chart review only revealed that he had passed away prior to completion of his treatment. The date of death was one year after surgical intervention, but the cause of death was unknown.

## Discussion

The first case of urothelial carcinoma metastasizing to the brain was reported by Lower and Watkins in 1924 [5,6,9]. Since then, reports of similar cases published in the literature have been few and far between. However, due to prolonged survival rates in urothelial carcinoma over the decades, the incidence of brain metastases may be increasing [4,9]. Historically, aggressive chemotherapeutic regimens have had poor central nervous system (CNS) penetrance [5]. Thus, the CNS may act as a central sanctuary site for distant metastases during peripheral systemic UC treatment [5]. Discussed later in this report, more recent chemotherapeutic regimens with blood-brain barrier penetrating potential may provide some relief for this situation [5].

While the exact underlying biological mechanisms of CNS metastases from UC have yet to be elucidated, it likely involves hematogenous spread through the Batson’s plexus [6]. Bladder cancer commonly spreads via local invasion as well as hematogenous and lymphatic dissemination [1,6,8]. Lymphatic dissemination can occur via obturator, superior and inferior gluteal, external and common iliac nodes [8]. From here, tumor cells may invade the para-aortic lymphatic chain and subsequently travel through the thoracic duct to enter general circulation [8]. In contrast, the hematogenous spread may occur via the pelvic plexus into the inferior vena cava or via paravertebral veins into the azygos, hemiazygos, intercostal, and other systemic veins [8]. In the case of brain metastases, invasion through the Batson venous plexus is strongly suspected [6]. Approximately 21% of patients with UC - and no concurrent evidence of extra-CNS metastases - will develop CNS metastasis [6]. Interestingly, those without extra-CNS disease had a shorter time to metastasis than those with extra-CNS metastasis [6]. Furthermore, 12-20% of patients are estimated to present with signs and symptoms of CNS metastasis without a known history of bladder cancer; some of these are later diagnosed with a UC primary [5,6]. These data suggest that a large proportion of patients seem to be predisposed towards rapid and insidious intracranial metastatic patterns. The nature of CNS metastasis from UC primary tumors thus poses a challenge for clinicians, emphasizing the importance of early recognition of signs and symptoms and consideration of the rare possibility of intracranial metastasis.

Urothelial carcinoma typically presents with gross hematuria and other irritative urinary symptoms [5]. Once metastasized to the brain, however, symptoms can include headache, dizziness, nausea and vomiting, speech impairment, cognitive impairment, and ataxia [2,5,10]. Fang et al. estimate that 20-40% of patients present with focal neurologic deficits, with hemiparesis being the primary form of impairment [10]. Similarly, Chandra et al. estimate that 20% of patients with isolated brain metastases from UC present with neurologic symptoms [2]. However, in either estimate, the majority of patients remain neurologically asymptomatic. When CNS involvement is suspected, testing for intracranial metastases and localization of lesions should be performed with a careful neurologic exam, followed by contrast-enhanced MRI, as CT has been reported to underestimate the number of intracranial metastases [1,10]. Following the identification of a potential metastatic lesion on imaging, there are several treatment options available for management.

In the late 20th century, the chemotherapeutic landscape for locally advanced bladder cancer was developing rapidly. Of the possible monotherapies, Cisplatin was found to be the most active agent [11]. However, many trials were underway to find the best combination therapy. The methotrexate-vinblastine-Adriamycin (doxorubicin)-cisplatin (MVAC) combination emerged at the top often in conjunction with partial or whole cystectomy [11]. Presently, gemcitabine and cisplatin (GC) are now considered the standard neoadjuvant therapy to cystectomy for UC as it shares a very similar efficacy to MVAC regimens and has a more palatable side effect profile for patients [12]. In rare cases such as in our patient, UC metastases spread to and penetrate the central nervous system (CNS) yielding a very poor prognosis [6]. Due to the lack of literature on the subject, the consensus on the mainstay treatment for metastatic UC to the brain remains anecdotal and mixed [6].

The challenge in treating advanced UC is that its metastatic potential is diffuse and aggressive [1,5,9]. Approximately 67% of patients with CNS metastases had previous non-CNS metastases from their bladder cancer and median survival was less than one year after diagnosis of metastatic brain cancer [6]. Thus, there are two main challenges when treating brain metastases from UC. The first is to establish local control and maintain remission of CNS metastases [9]. The second is to control the systemic disease of recurrent UC or non-CNS metastases [9]. To achieve local control, commonly used therapeutic treatments for brain metastases from bladder cancer include metastasectomy (debulking or complete resection) and adjuvant radiotherapy (stereotactic radiosurgery or whole brain radiotherapy) [6]. Most patients with multiple brain lesions with a more diffuse presentation receive whole brain radiotherapy (WBRT) with 10 x 3 Gy/2 weeks or 20 x 2 Gy/4 weeks as well as potentially including adjuvant chemotherapy [4,6]. Alternatively, patients with fewer and more localized metastases may opt for more targeted stereotactic radiosurgery (SRS) with partial or complete metastasectomy [4]. Unsurprisingly, patients with multiple metastases have a worse prognosis than those with a single metastasis [9,13]. The method to achieve systemic control is not clearly defined, however, some opt for additional adjuvant chemotherapy such as those that are gemcitabine-based [9]. After initial radiotherapy, gemcitabine monochemotherapy has shown to be a well-tolerated and effective treatment for slowing the progression of systemic burden and improving poor performance status [9]. This could be particularly good when the goal of therapy is palliative, and quality of life supersedes lengthening overall survival. Gemcitabine is of particular interest due to its ability to cross the blood-brain barrier and potentially aid in delaying cerebral metastases from primary UC [5]. Lastly, gemcitabine is also being investigated as a radiosensitizer in various other forms of brain cancer such as glioblastoma multiforme (GBM), making it a possible candidate to aid in local and systemic control of metastatic UC [14]. However, given the lack of robust clinical trials exploring chemotherapeutic, radiotherapeutic, and surgical intervention specifically in cases of UC with primary CNS metastasis, unequivocal treatment recommendations cannot confidently be made.

In this case, the patient presented with two intracranial metastatic lesions and no evidence of extracranial metastases. His initial diagnosis of urothelial carcinoma was treated per standard therapy with gemcitabine and cisplatin neoadjuvant chemotherapy in conjunction with partial cystectomy. On his recent presentation with intracranial metastases, the decision to resect the left-frontal tumor was made due to the need for local control, the need for a tissue biopsy for confirmation of pathology, and the need to improve the patient’s worsening neurologic symptoms in conjunction with his midline shift. Open resection of the right cerebellar lesion was not deemed necessary, and the therapeutic plan agreed upon was to pursue stereotactic radiosurgery. Additional treatment recommendations deferred to his medical oncologist.

## Conclusions

Distant metastases of urothelial carcinoma should always be considered a possibility. Although there is insufficient evidence to recommend routine imaging for intracranial metastases, clinicians should be aware of the increasing incidence of CNS metastases as more effective treatments develop to control primary disease and prolong life. In patients with a history of UC who complain of new neurologic deficits, a careful and thorough neurologic examination should be performed to help localize the lesion. Once identified, a contrast-enhanced MRI should be obtained to confirm the diagnosis. If lesions are identified, treatment options may include a combination of chemotherapy, radiation, and surgery. These should be discussed and decided upon within the context of prognostication and palliation.
